# Syntheses of Nickel (II) Complexes from Novel Semicarbazone Ligands with Chloroformylarylhydrazine, Benzimidazole and Salicylaldehyde Moieties

**DOI:** 10.3390/molecules20035184

**Published:** 2015-03-20

**Authors:** Mei-Hsiu Shih, Yu-Yuan Xu, Yu-Sheng Yang, Tzu-Ting Lin

**Affiliations:** Department of Chemical and Materials Engineering, Southern Taiwan University of Science and Technology, Tainan City 71005, Taiwan; E-Mails: ball3345678@yahoo.com.tw (Y.-Y.X.); yyyyyiyyyyy00@yahoo.com.tw (Y.-S.Y.); albee.lin@ekay.com.tw (T.-T.L.)

**Keywords:** α-chloroformylarylhydrazine, benzimidazole, salicylaldehyde, semicarbazides, semicarbazones, nickel complexes

## Abstract

This study addressed the design and syntheses of diverse ligands, which were then successfully treated with Ni (II) ion to afford a series of nickel complexes. α-Chloroformylarylhydrazine hydrochlorides **6** contain two different functional groups. One is a strong nucleophile, and the other is a good electrophile. Therefore, it can be designed to react with several reagents to obtain diverse derivatives which can be used as ligands for metal complexes. Furthermore, benzimidazole and salicylaldehyde can provide electron donor sites, *N* and *O* electron donors, separately. Hence, the starting materials α-chloroformylarylhydrazine hydrochlorides **6** were first treated with 2-(aminomethyl)-benzimidazole (**7**) to give the corresponding semicarbazides **8**. Then, the semicarbazides **8** reacted with various substituted salicylaldehydes **9**–**11** to afford the desired substituted-salicylaldehyde 2-aryl-4-substituted semicarbazones **12**–**14**, which could coordinate with nickel (II) ion to give the corresponding nickel complexes **15**–**17**.

## 1. Introduction

**Scheme 1:**
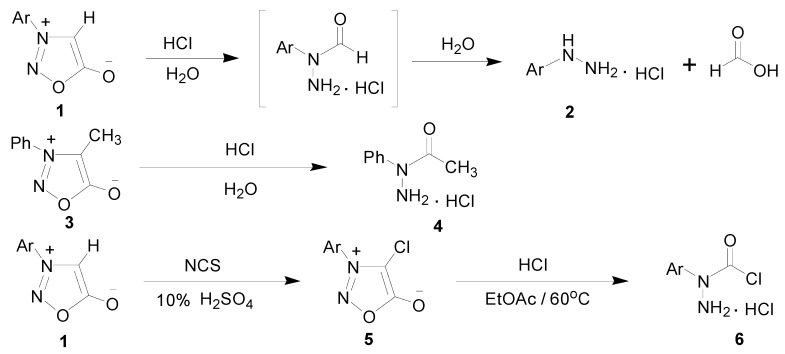
The preparation of α-chloroformylarylhydrazine hydrochlorides **6** from **3**-aryl-4-chlorosydnones **5**.

The design and synthesis of metal-organic frameworks have attracted much attention from chemists. Thiosemicarbazones, semicarbazones and their metal complexes have been extensively studied in recent years, mainly because of their potential biological properties [1,2,3]. However, less attention has been devoted to the synthesis of the structurally analogous semicarbazones and their metal complexes. Semicarbazones are readily available and can coordinate to the metal ion either as neutral or deprotonated ligands through two or three donor atoms. In order to obtain novel ligands containing semicarbazone moieties, adequate precursors should first be designed and investigated. 3-Arylsydnones **1** could be cleaved and hydrolyzed to α-formylarylhydrazine hydrochloride intermediates by hydrochloric acid, and then the intermediates were sequentially converted to 3-arylhydrazine hydrochlorides **2** [4]. However, 3-phenyl-4-methylsydnone (**3**) was only hydrolyzed to α-acetylphenylhydrazine (**4**) by hydrochloric acid [5]. According to the above result, we considered treating 3-arylsydnones **1** with *N*-chlorosuccinimde (NCS) to obtain 3-aryl-4-chlorosydnones **5** which could further react with hydrochloric acid to afford α-chloroformylarylhydrazine hydrochlorides **6**, as shown in Scheme 1. The precursors **6** would be expected to react with appropriate amines and aldehydes to afford various Schiff-bases which contained the desired semicarbazone moieties.

α-Chloroformylarylhydrazine hydrochlorides **6** contained two different functional groups. One is a very strong nucleophile, and the other is a good electrophile [6]. Precursors **6** could be treated with several reagents to give diverse derivatives [7]. We have already done abundant research on numerous aspects of 3-arylsydnone derivatives [8,9,10,11,12,13,14,15], and this is the first work conducted to utilize the starting materials **6**, which are derived from the decomposition of sydnone compounds, to synthesize diverse ligands and transition metal complexes.

## 2. Results and Discussion

### 2.1. Synthetic Chemistry

**Figure 1:**
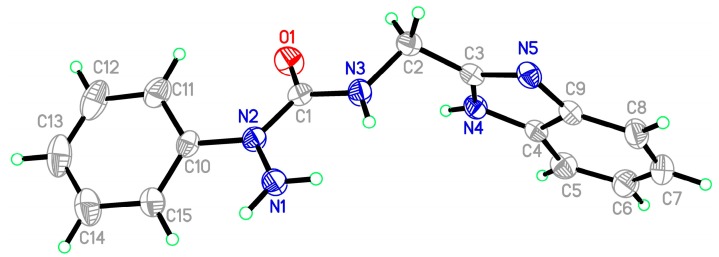
ORTEP drawing of 2-phenyl-4-[(1*H*-benzo[*d*]imidazol-2-yl)methyl]semicarbazide (**8a**).

**Figure 2:**
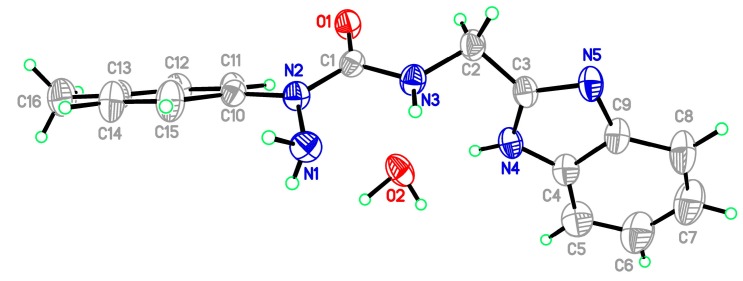
ORTEP drawing of 2-(4-methylphenyl)-4-[(1*H*-benzo[*d*]imidazol-2-yl)methyl]-semicarbazide (**8b**).

**Scheme 2:**
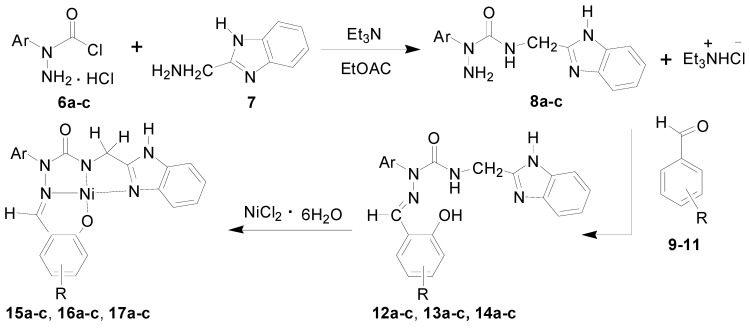
Novel **N**ickel (II) complexes **15**–**17** derived from diverse semicarbazone ligands **12**–**14**. **a**: Ar = C_6_H_5_; **b**: Ar = *p*-CH_3_C_6_H_4_; **c**: Ar = *p*-CH_3_OC_6_H_4_; **9**, **12**, **15**: R = H; **10**, **13**, **16**: R = 5-Cl; **11**, **14**, **17**: R = 4-OCH.

Starting materials **6**, since they contain acyl chloride groups, are very active and moisture-sensitive. We thus first dealt with the acyl chloride group of precursors **6** in the novel ligands design, and then dealt with the amino group to obtain the desired ligands. The benzimidazole scaffold is a useful structural motif for imparting chemical functionality to biologically active molecules [16,17,18,19,20], and many metal complexes with benzimidazole moieties display a wide range of special activities [21,22,23]. 2-(Aminomethyl)benzimidazole (**7**) can provide *N*,*N* two electron donor atoms, therefore, the starting materials **6a**–**c** were first treated with benzimidazole **7** at 0 °C in the presence of triethylamine to give the corresponding 2-aryl-4-[(1*H*-benzo[*d*]imidazol-2-yl)methyl]semicarbazides **8a**–**c**, as shown in Scheme 2. Because the starting materials **6** have a tendency to dimerize, the desired reactions must be carried out at 0 °C, otherwise, the efforts would fail. All the semicarbazides were synthesized in good yields and analytically pure. Among them, single crystals of **8a** and **8b** were suitable for X-ray structural analyses. Figure 1 and Figure 2 display the ORTEP drawings of semicarbazides **8a** and **8b**. Based on the X-ray data, semicarbazide **8b** was crystallized with a water molecule, and formed hydrogen bonds between the N_4_ atom and H_2_O because the N_4_ and O_2_ distance was 2.724 Å.

It was considered that the novel semicarbazides could be reacted with various salicylaldehydes to form the well-known hydrazone Schiff bases. Salicylaldehyde hydrazone Schiff base have long received considerable attention for their fascinating chemical behavior and biological activity. Of interest to chemists is the coordination ability of salicylaldehyde hydrazone ligand through the imine nitrogen and phenoxy oxygen electron-donating atoms that allow it to serve as a multidentate ligand in structural assemblies [24,25,26,27,28,29]. In this study, the synthesized semicarbazides **8a**–**c** were treated with various substituted salicylaldehydes, such as salicylaldehyde (**9**), 5-chlorosalicylaldehyde (**10**) and 4-methoxysalicylaldehyde (**11**) to afford a series of corresponding substituted-salicylaldehyde 2-aryl-4-[(1*H*-benzo[*d*]imidazol-2-yl)methyl]semicarbazones **12a**–**14c**. The semicarbazones **12a**–**14c** with salicylaldehyde-acylhydrazone moieties could coordinate with nickel (II) metal ion to give novel transition nickel (II) complexes **15a**–**17c** as shown in Scheme 2. The novel synthesized ligands **12**–**14** and metal complexes **15**–**17** were identified by IR, NMR, ESIMS, EA and X-ray crystallography.

### 2.2. Spectroscopy Studies of Ligands and Metal Complexes

#### 2.2.1. IR and NMR Studies

**Figure 3:**
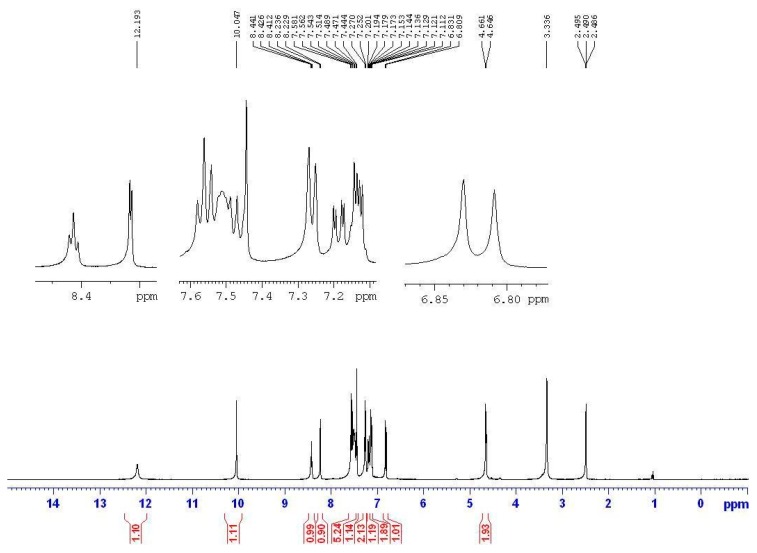
The NMR spectrum of semicarbazone **13a**.

**Figure 4:**
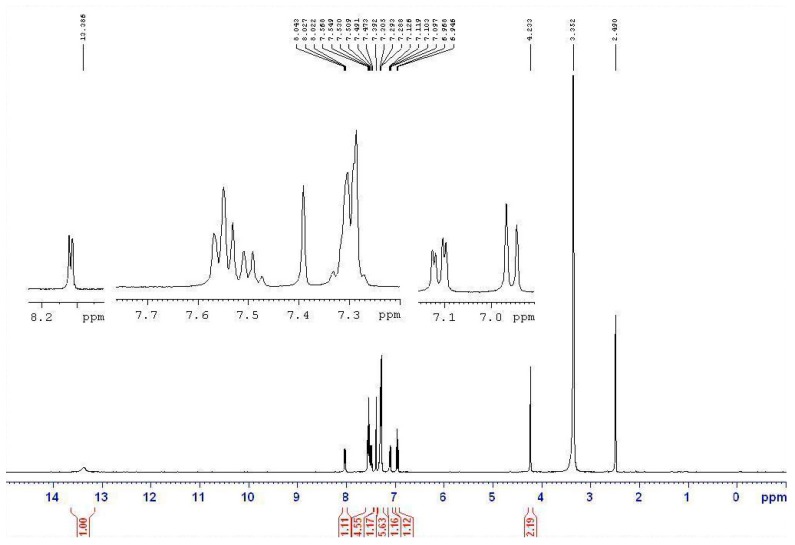
The NMR spectrum of nickel complex **16a** derived from semicarbazone **13a**.

Based on the IR studies, the O-H and N-H stretching frequencies observed at around 3400 and 3200 cm^−1^ in free semicarbazones **12**–**14** are found to be absent in the complexes **15**–**17**. The result confirms the deprotonation of these ligands upon metal complexation. Similarly, in the NMR studies, the signals at approximately 8.4 ppm (triplet, *J* = 5.6 Hz, 1H, NH) and about 10.0 ppm (singlet, 1H, OH) were originally assigned to the NH and OH protons of the semicarbazone. However, the signals are not found in the spectra of the nickel complexes **15**–**17**. In addition, the NMR signal of CH_2_ in the semicarbazones is at about 4.6 ppm (doublet, *J* = 5.6 Hz, 2H), and is split into a doublet by coupling with the neighboring NH proton. However, the NMR signal of CH_2_ is a singlet without any neighboring NH proton coupling in the metal complexes. Figure 3 and Figure 4 show the NMR spectrum of ligand **13a** and the corresponding complex **16a**, respectively. All the above results indicated that semicarbazone ligands **12**–**14** served as deprotoned ligands after losing two protons from the NH and OH groups upon metal complexation, and 2-(aminomethyl)benzimidazole (**7**) and substituted-salicylaldehydes **9**–**11** might provide electron donor sites, *N*, *N* and *O* electron donor atoms.

#### 2.2.2. MS Study

To further confirm the molecular formula of nickel complexes **15**–**17**, Fourier-Transfer Mass Analyzer was used to get the low resolution and high resolution ESI mass spectral data. The obtained analysis results definitely confirmed the molecular formulas of the synthesized complexes **15**–**17**. The low-resolution ESIMS spectra of all nickel complexes **15a**–**c** and **17a**–**c** show distinct *m*/*z* [M+H]^+^ and [M+2+H]^+^ peak patterns because the relative isotope abundances of elemental nickel are ^58^Ni (68.0769%), ^60^Ni (26.2231%), ^61^Ni (1.1399%), ^62^Ni (3.6435%) and ^64^Ni (0.9256%). Besides, the relative isotope abundances of the element chlorine are ^35^Cl (75.76%) and ^37^Cl (24.24%), nickel complexes **16a**–**c** containing one chlorine element would show more complicated MS peak patterns.

#### 2.2.3. X-ray Study of Ligands and Complexes

**Figure 5:**
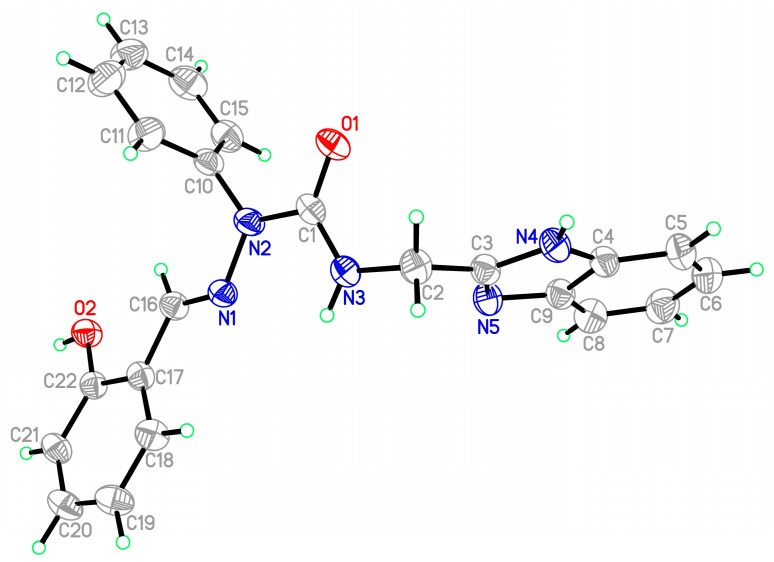
ORTEP drawing of salicylaldehyde 2-phenyl-4-[(1*H*-benzo[*d*]imidazol-2-yl)methyl]semicarbazone (**12a**).

**Figure 6:**
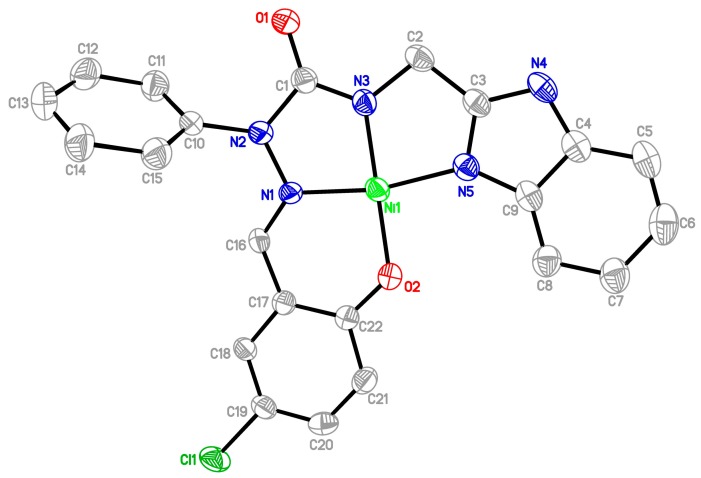
ORTEP drawing of Ni complex of 5-chlorosalicylaldehyde 2-phenyl-4-[(1*H*-benzo[*d*]imidazol-2-yl)methyl]semicarbazone (**16a**).

All the new semicarbazones and complexes were synthesized in good yields and analytically pure. Among them, the crystals of **12a** and **16a** were suitable for X-ray structure analyses. Figure 5 and Figure 6 show the ORTEP drawings of semicarbazone **12a**, and Ni complex **16a**, respectively. Based on the ORTEP drawing of semicarbazone **12a** (Figure 5), we confirm that the electron donor sites *N(1)*, *N(3)*, *N(5)* of free semicarbazone ligands **12**–**14** show a *Z*, *Z* configuration about N(2)-C(1), C(2)-C(3), and the hydroxyl oxygen is *anti* to the imine nitrogen *N(1)*. However, the nickel complex **16a** was crystallized with the salicylaldehyde hydroxyl oxygen *syn* to the imine nitrogen, as shown in Figure 6. According to the X-ray diffraction analyses, during metal complexation, semicarbazones **12**–**14** behave as tetradentate and deprotonated ligands after losing two protons from the OH and NH groups (Scheme 2), and form one six- and two five-membered chelate rings around the central metal through a set of donor atoms that consists of the salicylaldehyde hydroxyl oxygen, imine nitrogen, and two nitrogens of 2-(aminomethyl)benzimidazole (Figure 6).

**Figure 7:**
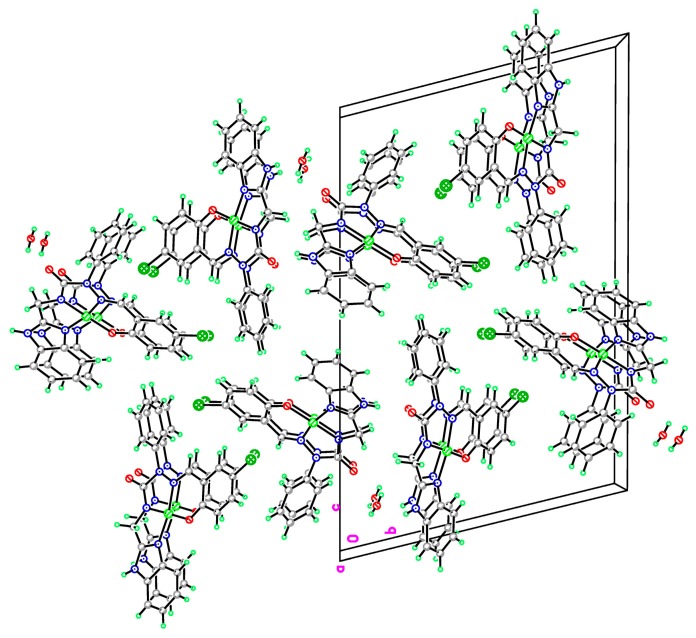
The packing diagram of complex **16a** crystallized with water molecule.

From the X-ray analysis of nickel complex **16a**, the bond lengths (Å) Ni-N(1), Ni-N(5), Ni-N(3), Ni-O(2) were 1.835(5), 1.885(5), 1.819(5), 1.834(4), and the bond angles (°) involving nickel N(3)-Ni-N(1), N(1)-Ni-O(2), N(3)-Ni-O(2), N(3)-Ni-N(5), N(5)-Ni-O(2), N(1)-Ni-N(5) were 83.5(2), 96.4(2), 178.8(2), 84.5(2), 95.6(2), 167.9(2). The result indicates that the tricyclic *N*,*N*,*N*,*O* ring system forms a nearly square planar structure around the nickel atom, and contributes to the stability of the complex **16a**. The ORTEP drawings of the metal complexes and diffraction data showed that the synthesized semicarbazones were tetradentate and dideprotoned ligands upon metal complexation. The nickel complex **16a** was crystallized with a water molecule, but there is no hydrogen bond between the N_4_ atom and H_2_O. Figure 7 displays the packing diagram of complex **16a**. The crystallographic data of semicarbazides **8a** and **8b** are summarized in Table 1. Table 2 lists the crystallographic data of semicarbazone **12a** and complex **16a**.

**Table 1:** Crystal data of semicarbazides **8a** and **8b**.

Compounds	8a	8b
Diffractometer	Nonius Kappa CCD	Nonius Kappa CCD
Formula	C_15_H_15_N_5_O	C_16_H_17_N_5_O·H_2_O
Formula weight	281.32	313.36
Crystal system	Monoclinic	Monoclinic
Space group	P2(1)/c	P2(1)/c
*a*/Å	14.0940(3)	15.3794(7)
*b*/Å	10.0209(2)	8.2222(5)
*c*/Å	10.1067(2)	13.8159(7)
α/°	90.00	90
β/°	104.088(2)	104.331(5)
γ/°	90.00	90
*V*/Å^3^	1384.48(5)	1692.69(15)
*Z*	4	4
*D*_calc_ (g·cm^−3^)	1.350	1.230
*F*_000_	592	664
Absorption coefficient (mm^−1^)	0.090	0.085
Crystal size/mm	0.25 × 0.20 × 0.15	0.25 × 0.25 × 0.15
Temperature (K)	295(2)	295(2)
θ_range_, deg	1.49–27.49	2.83–27.50
Reflections collected	18599	19743
Independent reflections	3161[*R*(int) = 0.0522]	3879 [*R*(int) = 0.0412]
Refinement method	Full-matrix least-squares on *F*^2^	Full-matrix least-squares on *F*^2^
Final *R* indices [*I* > 2.00σ(*I*)]	*R*1 = 0.0595, *wR*2 = 0.1625	*R*1 = 0.0828, *wR*2 = 0.2736
*R* indices (all data)	*R*1 = 0.0947, *wR*2 = 0.1888	*R*1 = 0.1071, *wR*2 = 0.2861
GoF	1.263	1.051

## 3. Experimental Section

### 3.1. General Information

All melting points were determined on an England Electrothermal Digital Melting Point apparatus and were uncorrected. IR spectra were recorded on a Mattson/Satellite 5000 FT-IR spectrophotometer. Mass spectra were measured on a high-resolution JEOL JMS-700 mass spectrometer and a Bruker APEX II FT-MS. ^1^H-NMR spectra were run on a Bruker AV 400 NMR spectrometer, using TMS as an internal standard. ^13^C-NMR spectra were recorded out with complete ^1^H decoupling and assignments were made through additional DEPT experiments. Elemental analyses were taken with an Elementar Vario EL-III Analyzer. X-ray crystallography was performed on a Nonius CAD4 Kappa Axis XRD instrument. α-Chloroformylarylhydrazine hydrochlorides **6a**–**c** were prepared from the corresponding 3-aryl-4-chlorosydnones **5a**–**c** according to the literature [6].

**Table 2:** Crystal data of semicarbazone **12a** and complex **16a**.

Compounds	12a	16a
Diffractometer	Nonius Kappa CCD	Nonius Kappa CCD
Formula	C_22_H_19_N_5_O_2_·C_2_H_5_OH	[Ni_2_(C_22_H_16_N_5_O_2_Cl)_2_]·H_2_O
Formula weight	431.49	971.13
Crystal system	Triclinic	Triclinic
Space group	P-1	P-1
*a*/Å	10.3571(7)	5.2360(4)
*b*/Å	10.3772(8)	16.6535(9)
*c*/Å	10.7518(7)	25.6203(16)
α/°	100.618(6)	75.822(5)
β/°	96.497(5)	88.784(6)
γ/°	90.357(6)	87.121(5)
*V*/Å^3^	1128.06(14)	2163.2(2)
*Z*	2	2
*D*_calc_ (g·cm^−3^)	1.270	1.491
*F*_000_	456	996
Absorption coefficient (mm^−1^)	0.700	1.052
Crystal size/mm	0.25 × 0.15 × 0.10	0.2 × 0.1 × 0.02
Temperature (K)	295(2)	295(2)
θ_range_, deg	4.30–67.93	3.03–27.50
Reflections collected	10692	17218
Independent reflections	4069 [*R*(int) = 0.0287]	9910 [*R*(int) = 0.0584]
Refinement method	Full-matrix least-squares on *F*^2^	Full-matrix least-squares on *F*^2^
Final *R* indices [*I* > 2.00σ(*I*)]	*R*1 = 0.0626, *wR*2 = 0.1871	*R*1 = 0.0898, *wR*2 = 0.2252
*R* indices (all data)	*R*1 = 0.0778, *wR*2 = 0.2127	*R*1 = 0.1419, *wR*2 = 0.2457
GoF	1.013	1.306

### 3.2. Syntheses of 2-aryl-4-[(1H-benzo[d]imidazol-2-yl)methyl]semicarbazides **8a**–**c**

To an ice-cooled solution of α-chloroformyl phenylhydrazine hydrochloride (**6a**, 207.1 mg, 1.0 mmol) in ethyl acetate (2 mL), an ice-cooled solution of 2-(aminomethyl)benzimidazole dihydrochloride hydrate (**7**, 242.2 mg, 1.1 mmol) in ethyl acetate (2 mL) was slowly added. Then, triethylamine (454.5 mg, 4.5 mmol) was added dropwise to the above solution. The mixed solution was stirred at 0 °C for about 6–7 h until the reaction was complete. The precipitating solid was first collected by filtration, and the organic filtrate was kept for the next step. First, the filtered solid was added to cold water (5 mL) with stirring and filtered to remove the dissolved triethylamine hydrochloride salt, then 261.5 mg of white crude product was obtained. Next, the organic filtrate was evaporated to near dryness and cold 2-propanol (1 mL) was added to precipitate a solid after stirring and filtration, and 21.2 mg of white solid were thus obtained. All the solid products were combined and recrystallized from dichloromethane/2-propanol to afford 221.9 mg (0.79 mmol, yield 79%) of **8a** as white crystals. The chemical and physical spectral characteristics of these products **8a**–**c** are given below.

*2-Phenyl-4-[(1H-benzo[d]imidazol-2-yl)methyl]semicarbazide* (**8a**): White crystals from CH_2_Cl_2_/(CH_3_)_2_CHOH; yield 79%; mp 172–174 °C; IR (KBr): 3343, 3327, 3283, 3198, 1649, 1597, 1519, 1417, 1291, 1178, 1011, 984, 837, 747 cm^−1^; ^1^H-NMR (DMSO-*d*_6_): δ 4.53 (d, *J* = 5.6 Hz, 2H, CH_2_), 5.28 (s, 2H, NH_2_), 7.02 (t, *J* = 8.0 Hz, 1H, ArH), 7.10–7.16 (m, 2H, imidazole-H), 7.28 (t, *J* = 8.0 Hz, 2H, ArH), 7.45–7.55 (m, 2H, imidazole-H), 7.62 (d, *J* = 8.0 Hz, 2H, ArH), 7.95 (t, *J* = 5.6 Hz, 1H, NH), 12.17 (s, 1H, NH); FABMS: *m*/*z* (%) = 282 ([M+H]^+^, 100), 281 (M^+^, 10), 174 (90), 131 (37). Anal. Calcd for C_15_H_15_N_5_O: C, 64.04; H, 5.37; N, 24.89. Found: C, 64.00; H, 5.35; N, 24.90. X-ray analytical data is listed in Table 1. Further details have been deposited at the Cambridge Crystallographic Data Center and allocated the deposition number CCDC 959866.

*2-(4-Methylphenyl)-4-[(1H-benzo[d]imidazol-2-yl)methyl]semicarbazide* (**8b**): White crystals from CH_2_Cl_2_/(CH_3_)_2_CHOH; yield 73%; mp 162–164 °C; IR (KBr): 3421, 3382, 3332, 3192, 1646, 1510, 1438, 1274, 1168, 1015, 996, 812, 742 cm^−1^; ^1^H-NMR (DMSO-*d*_6_): δ 2.25 (s, 3H, CH_3_), 4.52 (d, *J* = 5.6 Hz, 2H, CH_2_), 5.22 (s, 2H, NH_2_), 7.08 (d, *J* = 8.4 Hz, 2H, ArH), 7.12–7.14 (m, 2H, imidazole-H), 7.48 (d, *J* = 8.4 Hz, 2H, ArH) 7.49–7.56 (m, 2H, imidazole-H), 7.88 (t, *J* = 5.6 Hz, 1H, NH), 12.16 (s, 1H, NH); FABMS: *m*/*z* (%) = 296 ([M+H]^+^, 100), 295 (M^+^, 5), 174 (84), 131 (45). Anal. Calcd for C_16_H_17_N_5_O·H_2_O: C, 61.33; H, 6.11; N, 22.35. Found: C, 61.28; H, 6.09; N, 22.40. X-ray analytical data is listed in Table 1. Further details have been deposited at the Cambridge Crystallographic Data Center and allocated the deposition number CCDC 959867.

*2-(4-Methoxyphenyl)-4-[(1H-benzo[d]imidazol-2-yl)methyl]semicarbazide* (**8c**): White crystals from CH_2_Cl_2_/(CH_3_)_2_CHOH; yield 74%; mp 168–169 °C; IR (KBr): 3369, 3292, 3252, 3192, 1642, 1454, 1245, 1274, 1170, 1033, 977, 833, 743 cm^−1^; ^1^H-NMR (DMSO-*d*_6_): δ 3.72 (s, 3H, CH_3_O), 4.51 (d, *J* = 5.6 Hz, 2H, CH_2_), 5.20 (s, 2H, NH_2_), 6.86 (d, *J* = 8.8 Hz, 2H, ArH), 7.11–7.18 (m, 2H, imidazole-H), 7.46 (d, *J* = 8.8 Hz, 2H, ArH), 7.49–7.54 (m, 2H, imidazole-H), 7.80 (t, *J* = 5.6 Hz, 1H, NH), 12.15 (s, 1H, NH); FABMS: *m*/*z* (%) = 312 ([M+H]^+^, 100), 311 (M^+^, 4), 174 (92), 131 (100); HRMS (FAB): *m*/*z* [M+H]^+^ calcd for C_16_H_18_N_5_O_2_: 312.1460; found: 312.1462. Anal. Calcd for C_16_H_17_N_5_O_2_: C, 61.72; H, 5.50; N, 22.49. Found: C, 61.61; H, 5.51; N, 22.35.

### 3.3. Syntheses of Substituted-Salicylaldehyde 2-Aryl-4-[(1H-benzo[d]imidazol-2-yl)methyl] semicarbazones **12a**–**14c**

To a solution of 2-phenyl-4-(1H-benzimidazole-2-ylmethyl)semicarbazide (**8a**, 562.6 mg, 2.0 mmol) in absolute ethanol (6 mL), salicylaldehyde (**9**, 268.7 mg, 2.2 mmol) was added. The mixed solution was stirred at room temperature for about 8 h until the reaction was complete. The precipitated white powder (679.9 mg) was collected by filtration and washed with cold hexane/ethyl acetate (3:1). The collected solid was recrystallized from dichloromethane/ethanol to afford 602.5 mg (1.56 mmol, yield 78%) of **12a** as white powder. The chemical and physical spectral characteristics of these products **12a**–**c**, **13a**–**c**, **14a**–**c** are given below.

*Salicylaldehyde 2-phenyl-4-[(1H-benzo[d]imidazol-2-yl)methyl]semicarbazone* (**12a**): White crystals from CH_2_Cl_2_/EtOH; yield 78%; mp 243–244 °C; IR (KBr): 3415, 3230, 3213, 3060, 1668, 1605, 1517, 1450, 1272, 748 cm^−1^; ^1^H-NMR (DMSO-*d*_6_): δ 4.65 (d, *J* = 5.6 Hz, 2H, CH_2_), 6.80–6.85 (m, 2H, salicyl-H), 7.09–7.21 (m, 3H, imidazole-H, salicyl-H), 7.26 (d, *J* = 7.6 Hz, 2H, ArH), 7.42–7.61 (m, 6H, imidazole-H, =C-H, 3ArH), 8.12 (d, *J* = 7.6 Hz, 1H, salicyl-H), 8.24 (t, *J* = 5.6 Hz, 1H, NH), 9.75 (s, 1H, OH), 12.19 (s, 1H, NH); FABMS: *m*/*z* (%) = 386 ([M+H]^+^, 100), 385 ((M^+^, 10), 212 (30), 174 (51); HRMS (FAB): *m*/*z* [M+H]^+^ calcd for C_22_H_20_N_5_O_2_: 386.1617; found: 386.1715. Anal. Calcd for C_22_H_19_N_5_O_2_: C, 68.56; H, 4.97; N, 18.17. Found: C, 68.46; H, 4.96; N, 18.13. X-ray analytical data is listed in Table 2. Further details have been deposited at the Cambridge Crystallographic Data Center and allocated the deposition number CCDC 959868.

*Salicylaldehyde 2-(4-methylphenyl)-4-[(1H-benzo[d]imidazol-2-yl)methyl]semicarbazone* (**12b**): White powder from CH_2_Cl_2_/EtOH; yield: 81%; mp 226–227 °C; IR (KBr): 3419, 3375, 3180, 3057, 2927, 1665, 1605, 1525, 1456, 1265, 746 cm^−1^; ^1^H-NMR (DMSO-*d*_6_): δ 2.38 (s, 3H,CH_3_), 4.63 (d, *J* = 6.0 Hz, 2H, CH_2_), 6.78–6.86 (m, 2H, salicy-H), 7.08–7.20 (m, 5H, ArH, imidazole-H, salicyl-H), 7.35 (d, *J* = 8.0 Hz, 2H, ArH), 7.44–7.55 (m, 3H, imidazole-H, =C-H), 8.11 (d, *J* = 8.0 Hz, 1H, salicyl-H), 8.21 (t, *J* = 6.0 Hz, 1H, NH), 9.77 (s, 1H, OH), 12.18 (s, 1H, NH); ^13^C-NMR (DMSO-*d*_6_): δ = 21.04, 57.99, 111.46, 116.12, 118.50, 119.40, 120.76, 121.23, 121.87, 126.61, 129.86, 130.62, 130.85, 133.88, 134.51, 135.17, 138.47, 143.34, 153.55, 155.56, 156.11; FABMS: *m*/*z* (%) = 400 ([M+H]^+^, 100), 399 (M^+^, 8), 226 (58), 174 (83); HRMS (FAB): *m*/*z* [M+H]^+^ calcd for C_23_H_22_N_5_O_2_: 400.1774; found: 400.1772. Anal. Calcd for C_23_H_21_N_5_O_2_: C, 69.16; H, 5.30; N, 17.53. Found: C, 69.06; H, 5.31; N, 17.56.

*Salicylaldehyde 2-(4-methoxyphenyl)-4-[(1H-benzo[d]imidazol-2-yl)methyl]semicarbazone* (**12c**): White powder from CH_2_Cl_2_/EtOH; yield: 80%; mp 202–203 °C; IR (KBr): 3418, 3357, 3183, 3061, 2935, 1664, 1606, 1524, 1447, 1253, 744 cm^−1^; ^1^H-NMR (DMSO-*d*_6_): δ 3.82 (s, 3H, CH_3_O), 4.63 (d, *J* = 6.0 Hz, 2H, CH_2_), 6.78–6.87 (m, 2H, salicyl-H), 7.09 (d, *J* = 8.4 Hz, 2H, ArH), 7.11–7.15 (m, 3H, imidazole-H, salicyl-H), 7.18 (d, *J* = 8.4 Hz, 2H, ArH), 7.43–7.55 (m, 3H, =C-H, imidazole-H,), 8.10 (d, *J* = 7.6 Hz, 1H, salicyl-H), 8.19 (t, *J* = 6.0 Hz, 1H, NH), 9.80 (s, 1H, OH), 12.18 (s, 1H, NH); FABMS: *m*/*z* (%) = 416 ([M+H]^+^, 100), 410 (M^+^, 4); HRMS (FAB): *m*/*z* [M+H]^+^ calcd for for C_23_H_22_N_5_O_3_: 416.1723; found: 416.1723. Anal. Calcd for C_23_H_21_N_5_O_3_: C, 66.49; H, 5.09; N, 16.86. Found: C, 66.40; H, 5.07; N, 16.81.

*5-Chlorosalicylaldehyde 2-phenyl-4-[(1H-benzo[d]imidazol-2-yl)methyl]semicarbazone* (**13a**): White powder from CH_2_Cl_2_/EtOH; yield 75%; mp 231–232 °C; IR (KBr): 3419, 3210, 3065, 2937, 1675, 1624, 1518, 1423, 1284, 1275, 1196, 1108, 1032, 814, 743, 696 cm^−1^; ^1^H-NMR (DMSO-*d*_6_): δ 4.65 (d, *J* = 5.6 Hz, 2H, CH_2_), 6.81 (d, *J* = 8.8 Hz, 1H, salicyl-H), 7.11–7.15 (m, 2H, imidazole-H), 7.18 (dd, *J* = 8.8, 2.8 Hz, 1H, salicyl-H), 7.26 (d, *J* = 7.6 Hz, 2H, ArH), 7.44–7.58 (m, 6H, 3ArH, imidazole-H, =C-H), 8.23 (d, *J* = 2.8 Hz, 1H, salicyl-H), 8.42 (t, *J* = 5.6 Hz, 1H, NH), 10.03 (s, 1H, OH), 12.18 (s, 1H, NH); MS (EI, 30ev): *m*/*z* (%) = 421 ([M+2]^+^, 2), 419 (M^+^, 6), 248 ([M+2-C_7_N_2_H_5_CH_2_NCO]^+^, 15), 246 (M^+^-C_7_N_2_H_5_CH_2_NCO, 46), 174 (C_7_N_2_H_5_CH_2_NHCO, 100) 173 (C_7_N_2_H_5_CH_2_NCO, 41); HRMS (EI): *m*/*z* [M]^+^ calcd for C_22_H_18_N_5_O_2_^35^Cl: 419.1149; found: 419.1152. Anal. Calcd for C_22_H_18_N_5_O_2_Cl: C, 62.93; H, 4.32; N, 16.68. Found: C, 62.82; H, 4.31; N, 16.72.

*5-Chlorosalicylaldehyde 2-(4-methylphenyl)-4-[(1H-benzo[d]imidazol-2-yl)methyl]semicarbazone* (**13b**): White powder from CH_2_Cl_2_/EtOH; yield 76%; mp 229–230 °C; IR (KBr): 3405, 3196, 3063, 2924, 1666, 1606, 1510, 1425, 1273, 1206, 1107, 1029, 820, 743, 655 cm^−1^; ^1^H-NMR (DMSO-*d*_6_): δ 2.38 (s, 3H, CH_3_), 4.64 (d, *J* = 5.6 Hz, 2H, CH_2_), 6.82 (d, *J* = 8.8 Hz, 1H, salicyl-H), 7.09–7.15 (m, 4H, ArH, imidazole-H), 7.18 (dd, *J* = 8.8, 2.4 Hz, 1H, salicyl-H), 7.35 (d, *J* = 8.0 Hz, 2H, ArH), 7.45–7.55 (m, 3H, imidazole-H, =C-H), 8.21 (d, *J* = 2.4 Hz, 1H, salicyl-H), 8.38 (t, *J* = 5.6 Hz, 1H, NH), 10.04 (s, 1H, OH), 12.17 (s, 1H, NH); MS (EI, 30ev): *m*/*z* (%) = 435 ([M+2]^+^, 2), 433 (M^+^, 5), 262 ([M+2-C_7_N_2_H_5_CH_2_NCO]^+^, 36), 260 (M^+^-C_7_N_2_H_5_CH_2_NCO, 100), 174 (C_7_N_2_H_5_CH_2_NHCO, 79) 173 (C_7_N_2_H_5_CH_2_NCO, 42); HRMS (EI): *m*/*z* [M]^+^ calcd for C_23_H_20_N_5_O_2_^35^Cl: 433.1306; found: 433.1308. Anal. Calcd for C_23_H_20_N_5_O_2_Cl: C, 63.67; H, 4.65; N, 16.14. Found: C, 63.65; H, 4.63; N, 16.10.

*5-Chlorosalicylaldehyde 2-(4-methoxyphenyl)-4-[(1H-benzo[d]imidazol-2-yl)methyl]semicarbazone* (**13c**): White powder from CH_2_Cl_2_/EtOH; yield 82%; mp 245–246 °C; IR (KBr): 3403, 3206, 3072, 2935, 1662, 1606, 1508, 1422, 1283, 1249, 1182, 1029, 821, 751, 653 cm^−1^; ^1^H-NMR (DMSO-*d*_6_): δ 3.81 (s, 3H, CH_3_O), 4.63 (d, *J* = 6.0 Hz, 2H, CH_2_), 6.82 (d, *J* = 8.8 Hz, 1H, salicyl-H), 7.05–7.20 (m, 7H, 4ArH, imidazole-H, salicyl-H), 7.42–7.58 (m, 3H, imidazole-H, =C-H), 8.21 (d, *J* = 2.4 Hz, 1H, salicyl-H), 8.36 (t, *J* = 6.0 Hz, 1H, NH), 10.04 (s, 1H, OH), 12.16 (s, 1H, NH); MS (EI, 30ev): *m*/*z* (%) = 451 ([M+2]^+^, 2), 449 (M^+^, 6), 278 ([M+2-C_7_N_2_H_5_CH_2_NCO]^+^, 39), 276 (M^+^-C_7_N_2_H_5_CH_2_NCO, 100), 174 (C_7_N_2_H_5_CH_2_NHCO, 71) 173 (C_7_N_2_H_5_CH_2_NCO, 38); HRMS (EI): *m*/*z* [M]^+^ calcd for C_23_H_20_N_5_O_3_^35^Cl: 449.1255; found: 449.1258. Anal. Calcd for C_23_H_20_N_5_O_3_Cl: C, 61.40; H, 4.48; N, 15.57. Found: C, 61.49; H, 4.46; N, 15.60.

*4-Methoxysalicylaldehyde 2-phenyl-4-[(1H-benzo[d]imidazol-2-yl)methyl]semicarbazone* (**14a**): White powder from CH_2_Cl_2_/EtOH; yield 80%; mp 218–219 °C; IR (KBr): 3417, 3231, 3019, 2942, 2870, 1666, 1614, 1523, 1436, 1292, 1202, 1031, 745 cm^−1^; ^1^H-NMR (DMSO-*d*_6_): δ 3.70 (s, 3H, OCH_3_), 4.62 (d, *J* = 6.0 Hz, 2H, CH_2_), 6.35 (d, *J* = 2.4 Hz, 1H, salicyl-H), 6.43 (dd, *J* = 8.8, 2.4 Hz, 1H, salicyl-H), 7.11–7.24 (m, 2H, imidazole-H), 7.25 (d, *J* = 7.2 Hz, 2H, ArH), 7.44–7.63 (m, 6H, 3ArH, imidazole-H, =C-H), 8.02 (d, *J* = 8.8 Hz, 1H, salicyl-H), 8.17 (t, *J* = 6.0 Hz, 1H, NH), 9.84 (s, 1H, OH), 12.17 (s, 1H, NH); MS (EI, 30ev): *m*/*z* (%) = 415 (M^+^, 2), 242 (M^+^-C_7_N_2_H_5_CH_2_NCO, 100), 173 (C_7_N_2_H_5_CH_2_NCO, 25); HRMS (EI): *m*/*z* [M]^+^ calcd for C_23_H_21_N_5_O_3_: 415.1644; found: 415.1642. Anal. Calcd for C_23_H_21_N_5_O_3_: C, 66.49; H, 5.09; N, 16.86. Found: C, 66.52; H, 5.08; N, 16.79.

*4-Methoxysalicylaldehyde* *2-(4-methylphenyl)-4-[(1H-benzo[d]imidazol-2-yl)methyl]semicarbazone* (**14b**): White powder from CH_2_Cl_2_/EtOH; yield 81%; mp 198–199 °C; IR (KBr): 3418, 3174, 3143, 3059, 2963, 1643, 1609, 1511, 1438, 1291, 1237, 1208, 1031, 740 cm^−1^; ^1^H-NMR (DMSO-*d*_6_): δ 2.37 (s, 3H, CH_3_), 3.71 (s, 3H, OCH_3_), 4.63 (d, *J* = 5.6 Hz, 2H, CH_2_), 6.36 (d, *J* = 2.4 Hz, 1H, salicyl-H), 6.43 (dd, *J* = 8.8, 2.4 Hz, 1H, salicyl-H), 7.10–7.14 (m, 4H, ArH, imidazole-H), 7.34 (d, *J* = 8.0 Hz, 2H, ArH), 7.45–7.60 (m, 3H, =C-H, imidazole-H), 8.00 (d, *J* = 8.8 Hz, 1H, salicyl-H), 8.12 (t, *J* = 5.6 Hz, 1H, NH), 9.88 (s, 1H, OH), 12.17 (s, 1H, NH); MS (EI, 30ev): *m*/*z* (%) = 429 (M^+^, 1), 256 (M^+^-C_7_N_2_H_5_CH_2_NCO, 100), 173 (C_7_N_2_H_5_CH_2_NCO, 37); HRMS (EI): *m*/*z* [M]^+^ calcd for C_24_H_23_N_5_O_3_: 429.1801; found: 429.1802. Anal. Calcd for C_24_H_23_N_5_O_3_: C, 67.12; H, 5.40; N, 16.31. Found: C, 67.01; H, 5.38; N, 16.34.

*4-Methoxysalicylaldehyde 2-(4-methoxyphenyl)-4-[(1H-benzo[d]imidazol-2-yl)methyl]semicarbazone* (**14c**): White powder from CH_2_Cl_2_/EtOH; yield 84%; mp 170–171 °C; IR (KBr): 3404, 3190, 3059, 2837, 1657, 1609, 1510, 1441, 1295, 1250, 1205, 1031, 743 cm^−1^; ^1^H-NMR (DMSO-*d*_6_): δ 3.70 (s, 3H, OCH_3_), 3.81 (s, 3H, CH_3_O), 4.61 (d, *J* = 5.6 Hz, 2H, CH_2_), 6.36 (d, *J* = 2.4 Hz, 1H, salicyl-H), 6.42 (dd, *J* = 8.8, 2.4 Hz, 1H, salicy-H), 7.05–7.20 (m, 6H, ArH, imidazole-H), 7.40–7.55 (m, 3H, imidazole-H, =C-H), 7.98 (d, *J* = 8.8 Hz, 1H, salicyl-H ), 8.09 (t, *J* = 5.6 Hz, 1H, NH), 9.92 (s, 1H, OH), 12.16 (s, 1H, NH); MS (EI, 30ev): *m*/*z* (%) = 445 (M^+^, 1), 272 (M^+^-C_7_N_2_H_5_CH_2_NCO, 100), 173 (C_7_N_2_H_5_CH_2_NCO, 23); HRMS (EI): *m*/*z* [M]^+^ calcd for C_24_H_23_N_5_O_4_: 445.1750; found: 445.1751. Anal. Calcd for C_24_H_23_N_5_O_4_: C, 64.71; H, 5.20; N, 15.72. Found: C, 64.66; H, 5.18; N, 15.79.

### 3.4. Syntheses of Ni Complexes of Substituted-salicylaldehyde 2-Aryl-4-[(1H-benzo[d]imidazol-2-yl)methyl] Semicarbazones **15a**–**17c**

To a hot solution of salicylaldehyde 2-phenyl-4-[(1*H*-benzo[*d*]imidazol-2-yl)methyl] semicarbazone (**12a**, 385.4 mg, 1.0 mmol) in 95% ethanol (8 mL, 60 °C), a green hot solution of nickel(II) chloride hexahydrate (356.6 mg, 1.5 mmol) in distilled water (4 mL, 60 °C) was added dropwise. The orange mixture appeared cloudy and some solid precipitated out. The mixture was still heated at 60 °C and stirred for about 4–5 days until the reaction was complete. The precipitated orange powder (298.5 mg) was collected by filtration and washed with cold water and cold ethanol. The collected powder was recrystallized from dichloromethane/ethanol to afford 264.5 mg (0.60 mmol, yield 60%) of **15a** as golden-yellow crystals. The chemical and physical spectral characteristics of the Ni complexes **15a**–**c**, **16a**–**c**, **17a**–**c** are given below.

*Ni complex of salicylaldehyde 2-phenyl-4-[(1H-benzo[d]imidazol-2-yl)methyl]semicarbazone* (**15a**): Golden-yellow crystals from CH_2_Cl_2_/EtOH; yield 60%; mp 221–223 (dec) °C; IR (KBr): 3183, 3056, 2917, 1658, 1603, 1455, 1365, 1317, 1205, 1150, 1108, 839, 738, 593 cm^−1^; ^1^H-NMR (DMSO-*d*_6_): δ 4.24 (s, 2H, CH_2_), 6.48 (t, *J* = 8.0 Hz, 1H, salicyl-H), 6.95 (d, *J* = 8.0 Hz, 1H, salicyl-H), 7.08–7.16 (m, 2H, imidazole-H), 7.26–7.40 (m, 5H, 2ArH, =C-H, salicyl-2H), 7.45–7.60 (m, 4H, 3ArH, imidazole-H), 8.06–8.12 (m, 1H, imidazole-H), 13.37 (s, 1H, NH); MS (ESI): *m*/*z* (%) = 442 ([M+H]^+^, 100), 444 ([M+2+H]^+^, 28); HRMS (ESI): *m*/*z* [M+H]^+^ calcd for ^58^NiC_22_H_18_N_5_O_2_: 442.0814; found: 442.0816. Anal. Calcd for C_22_H_17_N_5_O_2_Ni: C, 59.77; H, 3.88; N, 15.84. Found: C, 59.68; H, 3.89; N, 15.79.

*Ni complex of salicylaldehyde 2-(4-methylphenyl)-4-[(1H-benzo[d]imidazol-2-yl)methyl]semicarbazone* (**15b**): Golden-yellow powder from CH_2_Cl_2_/EtOH; yield 66%; mp 242–244 (dec) °C; IR (KBr): 3167, 3024, 2910, 2839, 1649, 1604, 1451, 1363, 1317, 1203, 1148, 1098, 813, 735, 589 cm^−1^; ^1^H-NMR (DMSO-*d*_6_): δ 2.38 (s, 3H, CH_3_), 4.23 (s, 2H, CH_2_), 6.47 (t, *J* = 8.0 Hz, 1H, salicy-H), 6.95 (d, *J* = 8.0 Hz, 1H, salicyl-H), 7.05–7.20 (m, 4H, 2ArH, imidazole-H), 7.29–7.36 (m, 5H, 2ArH, =C-H, salicyl-H), 7.56 (d, *J* = 5.6 Hz, 1H, imidazole-H), 8.09 (d, *J* = 5.6 Hz, 1H, imidazole-H), 13.36 (s, 1H, NH); MS (ESI): *m*/*z* (%) = 456 ([M+H]^+^, 100), 458 ([M+2+H]^+^, 32); HRMS (ESI): *m*/*z* [M+H]^+^ calcd for ^58^NiC_23_H_20_N_5_O_2_: 456.0970; found: 456.0973. Anal. Calcd for C_23_H_19_N_5_O_2_Ni: C, 60.57; H, 4.20; N, 15.35. Found: C, 60.48; H, 4.19; N, 15.36.

*Ni complex of salicylaldehyde 2-(4-methoxyphenyl)-4-[(1H-benzo[d]imidazol-2-yl)methyl]semicarbazone* **(15c**): Orange-red crystals from CH_2_Cl_2_/EtOH; yield 62%; mp 219–221 (dec) °C; IR (KBr): 3113, 3034, 2963, 2907, 2837, 1650, 1605, 1448, 1364, 1315, 1248, 1204, 1148, 1092, 821, 735, 589 cm^−1^; ^1^H-NMR (DMSO-*d*_6_): δ 3.82 (s, 3H, CH_3_O), 4.23 (s, 2H, CH_2_), 6.47 (t, *J* = 8.0 Hz, 1H, salicyl-H), 6.95 (d, *J* = 8.0 Hz, 1H, salicyl-H), 7.05–7.16 (m, 4H, 2ArH, imidazole-H), 7.23 (d, *J* = 8.8 Hz, 2H, ArH), 7.29–7.36 (m, 3H, salicyl-H, =C-H), 7.56 (dd, *J* = 6.4, 2.4 Hz, 1H, imidazole-H), 8.09 (dd, *J* = 6.4, 2.4 Hz, 1H, imidazole-H), 13.37 (s, 1H); MS (ESI): *m*/*z* (%) = 472 ([M+H]^+^, 100), 474 ([M+2+H]^+^, 35); HRMS (ESI): *m*/*z* [M+H]^+^ for ^58^NiC_23_H_20_N_5_O_3_: 472.0920; found: 472.0923. Anal. Calcd for C_23_H_19_N_5_O_3_Ni: C, 58.51; H, 4.06; N, 14.83. Found: C, 58.46; H, 4.05; N, 14.79.

*Ni complex of 5-chlorosalicylaldehyde 2-phenyl-4-[(1H-benzo[d]imidazol-2-yl)methyl]semicarbazone* (**16a**): Orange-red needles from CH_2_Cl_2_/EtOH; yield 80%; mp 341–343 (dec) °C; IR (KBr): 3117, 3059, 2894, 1663, 1593, 1536, 1465, 1433, 1369, 1312, 1292, 1184, 1047, 813, 756, 698, 601 cm^−1^; ^1^H-NMR (DMSO-*d*_6_): δ 4.24 (s, 2H, CH_2_), 6.96 (d, *J* = 8.8 Hz, 1H, salicyl-H), 7.10 (dd, *J* = 8.8, 2.8 Hz, 1H, salicyl-H), 7.28–7.34 (m, 5H, 2ArH, imidazole-2H, salicyl-H), 7.40 (s, 1H, =C-H), 7.47–7.57 (m, 4H, 3ArH, imidazole-H), 8.03–8.05 (m, 1H, imidazole-H), 13.38 (s, 1H, NH); MS (ESI): *m*/*z* (%) = 476 ([M+H]^+^, 100), 478 ([M+2+H]^+^, 55); HRMS (ESI): *m*/*z* [M+H]^+^ for ^58^NiC_22_H_17_N_5_O_2_^35^Cl: 476.0424; found: 476.0425. Anal. Calcd for C_22_H_16_N_5_O_2_ClNi: C, 55.45; H, 3. 39; N, 14.70. Found: C, 55.48; H, 3.40; N, 14.74. X-ray analytical data is listed in Table 2. Further details have been deposited at the Cambridge Crystallographic Data Center and allocated the deposition number CCDC 959869.

*Ni* *complex of 5-chlorosalicylaldehyde 2-(4-methylphenyl)-4-[(1H-benzo[d]imidazol-2-yl)methyl]semicarbazone* (**16b**): Orange powder from CH_2_Cl_2_/EtOH; yield 62%; mp 362–364 (dec) °C; IR (KBr): 3107, 3050, 2916, 1661, 1592, 1537, 1461, 1434, 1367, 1293, 1183, 1045, 814, 754, 598 cm^−1^; ^1^H-NMR (DMSO-*d*_6_): δ 2.38 (s, 3H, CH_3_), 4.24 (s, 2H, CH_2_), 6.96 (d, *J* = 9.2 Hz, 1H, salicyl-H), 7.11 (dd, *J* = 9.2, 2.8 Hz, 1H, salicyl-H), 7.17 (d, *J* = 8.4 Hz, 2H, ArH), 7.29–7.36 (m, 5H, salicyl-H, imidazole-2H, 2ArH), 7.39 (s, 1H, =C-H), 7.54–7.59 (m, 1H, imidazole-H), 8.01–8.07 (m, 1H, imidazole-H), 13.37 (s, 1H, NH); MS (ESI): *m*/*z* (%) = 490 ([M+H]^+^, 100), 492 ([M+2+H]^+^, 65); HRMS (ESI): *m*/*z* [M+H]^+^ for ^58^NiC_23_H_19_N_5_O_2_^35^Cl: 490.0581; found: 490.0582. Anal. Calcd for C_2__3_H_1__8_N_5_O_2_ClNi: C, 56.31; H, 3.70; N, 14.28. Found: C, 56.22; H, 3.71; N, 14.24.

*Ni complex of 5-chlorosalicylaldehyde 2-(4-methoxyphenyl)-4-[(1H-benzo[d]imidazol-2-yl)methyl]semicarbazone* (**16c**): Orange feathers from CHCl_3_/EtOH; yield 60%; mp 383–385 (dec) °C; IR (KBr): 3103, 3048, 2989, 2888, 1659, 1593, 1512, 1462, 1435, 1366, 1308, 1255, 1182, 1046, 816, 753, 664, 599 cm^−1^; ^1^H-NMR (DMSO-*d*_6_): δ 3.84 (s, 3H, CH_3_O), 4.25 (s, 2H, CH_3_), 6.98 (d, *J* = 8.8 Hz, 1H, salicyl-H), 7.08–7.14 (m, 3H, 2ArH, salicyl-H), 7.23 (d, *J* = 8.8 Hz, 2H, ArH), 7.31–7.34 (m, 2H, imidazole-2H), 7.35 (d, *J* = 2.8 Hz, 1H, salicyl-H), 7.39 (s, 1H, =C-H), 7.55–7.61 (m, 1H, imidazole-H), 8.03–8.09 (m, 1H, imidazole-H), 13.40 (s, 1H, NH); MS (ESI): *m*/*z* (%) = 506 ([M+H]^+^, 100), 508 ([M+2+H]^+^, 60); HRMS (ESI): *m*/*z* [M+H]^+^ for ^58^NiC_23_H_19_N_5_O_3_^35^Cl: 506.0530; found: 506.0533. Anal. Calcd for C_2__3_H_1__8_N_5_O_3_ClNi: C, 54.53; H, 3.58; N, 13.82. Found: C, 54.42; H,3.57; N, 13.79.

*Ni complex of 4-methoxysalicylaldehyde 2-phenyl-4-[(1H-benzo[d]imidazol-2-yl)methyl]semicarbazone* (**17a**): Orange-red needles from CHCl_3_/EtOH; yield 68%; mp 325–326 (dec) °C; IR (KBr): 3181, 3051, 2911, 2840, 1642, 1604, 1435, 1370, 1218, 1126, 1049, 826, 724, 539 cm^−1^; ^1^H-NMR (DMSO-*d*_6_): δ 3.74 (s, 3H, OCH_3_), 4.22 (s, 2H, CH_2_), 6.14 (dd, *J* = 8.8, 2.4 Hz, 1H, salicyl-H), 6.46 (d, *J* = 2.4 Hz, 1H, salicyl-H), 7.01 (d, *J* = 8.8 Hz, 1H, salicyl-H), 7.26 (s, 1H, =C-H), 7.28–7.34 (m, 4H, 2ArH, imidazole-2H), 7.44–7.58 (m, 4H, 3ArH, imidazole-H), 8.08 (dd, *J* = 6.0, 3.2 Hz, 1H, imidazole-H), 13.35 (s, 1H, NH); MS (ESI): *m*/*z* (%) = 472 ([M+H]^+^, 100), 474 ([M+2+H]^+^, 30); HRMS (ESI): *m*/*z* [M+H]^+^ for ^58^NiC_23_H_20_N_5_O_3_: 472.0920; found: 472.0917. Anal. Calcd for C_2__3_H_1__9_N_5_O_3_Ni: C, 58.51; H, 4.06; N, 14.83. Found: C, 58.46; H, 4.04; N, 14.76.

*Ni complex of 4-methoxysalicylaldehyde 2-(4-methylphenyl)-4-[(1H-benzo[d]imidazol-2-yl)methyl]semicarbazone* (**17b**): Orange-red needles from CHCl_3_/EtOH; yield 63%; mp 318–319 (dec) °C; IR (KBr): 3186, 3066, 2914, 2834, 1643, 1606, 1438, 1372, 1221, 1118, 1038, 827, 745, 537 cm^−1^; ^1^H-NMR (DMSO-*d*_6_): δ 2.37 (s, 3H, CH_3_), 3.74 (s, 3H, OCH_3_), 4.21 (s, 2H, CH_2_), 6.13 (d, *J* = 8.8 Hz, 1H, salicyl-H), 6.45 (s, 1H, salicyl-H), 7.01 (d, *J* = 8.8 Hz, 1H, salicyl-H), 7.16 (d, *J* = 7.6 Hz, 2H, ArH), 7.23 (s, 1H, =C-H), 7.28–7.40 (m, 4H, 2ArH, imidazole-H), 7.56 (brs, 1H, imidazole-H), 8.07 (brs, 1H, imidazole-H), 13.35 (s, 1H, NH); MS (ESI): *m*/*z* (%) = 486 ([M+H]^+^, 100), 488 ([M+2+H]^+^, 35); HRMS (ESI): *m*/*z* [M+H]^+^ for ^58^NiC_24_H_22_N_5_O_3_: 486.1076; found: 486.1078. Anal. Calcd for C_2__4_H_21_N_5_O_3_Ni: C, 59.30; H, 4.35; N, 14.41. Found: C, 59.18; H, 4.37; N, 14.35.

*Ni complex of 4-methoxysalicylaldehyde* *2-(4-methoxyphenyl)-4-[(1H-benzo[d]imidazol-2-yl)methyl]semicarbazone* (**17c**): Yellow powder from CH_2_Cl_2_/EtOH; yield 64%; mp 335–336 (dec) °C; IR (KBr): 3118, 3051, 2905, 2836, 1654, 1606, 1440, 1372, 1219, 1105, 1043, 824, 744, 542 cm^−1^; ^1^H-NMR (DMSO-*d*_6_): δ 3.74 (s, 3H, OCH_3_), 3.81 (s, 3H, CH_3_O), 4.21 (s, 2H, CH_2_), 6.13 (dd, *J* = 8.8, 1.2 Hz, 1H, salicyl-H), 6.44 (d, *J* = 1.2 Hz, 1H, salicyl-H), 7.01 (d, *J* = 8.8 Hz, 1H, salicyl-H), 7.06 (d, *J* = 8.8 Hz, 2H, ArH), 7.14–7.21 (m, 3H, 2ArH, =C-H), 7.30–7.32 (m, 2H, imidazole-H), 7.56 (dd, *J* = 6.0, 2.8 Hz, 1H, imidazole-H), 8.07 (dd, *J* = 6.0, 2.8 Hz, 1H, imidazole-H), 13.34 (s, 1H, NH); MS (ESI): *m*/*z* (%) = 502 ([M+H]^+^, 100), 504 ([M+2+H]^+^, 30); HRMS (ESI): *m*/*z* [M+H]^+^ for ^58^NiC_24_H_22_N_5_O_4_: 502.1025; found: 502.1023. Anal. Calcd for C_2__4_H_21_N_5_O_4_Ni: C, 57.41; H, 4.22; N, 13.95. Found: C, 57.45; H, 4.23; N, 13.92.

## 4. Conclusions

In conclusion, this is the first work conducted to investigate the coordination chemistry of novel ligands containing the α-chloroformylarylhydrazine, benzimidazole and salicylaldehyde moieties. α-Chloroformylarylhydrazine hydrochlorides **6** were obtained through ring opening of 3-aryl-4-chlorosydnones with conc. hydrochloric acid. The starting materials **6** contained two special functional groups and had been proved to be good potential precursors of important ligands for metal complex formation in the study. The starting materials **6** were first treated with 2-(aminomethyl)benzimidazole (**7**) to give the corresponding semicarbazides **8**. Then, the semicarbazides **8** reacted with various substituted salicylaldehydes **9**–**11** to afford the corresponding semicarbazones **12**–**14** which could coordinate with nickel (II) metal ions in a *N*,*N*,*N*,*O-*tetradentate coordination mode. Novel synthesized ligands and complexes were characterized by IR, NMR, ESIMS, EA and X-ray diffraction. Based on the analytical results, the most reasonable structure for the nickel complexes is nearly square planar. Semicarbazones **12**–**14** behave as tetradentate and deprotonated ligands after losing two protons from the OH and NH groups, and form one six- and two five-membered chelate rings around the central nickel metal through a set of donor atoms that consists of the salicylaldehyde hydroxyl oxygen, imine nitrogen, and two nitrogens of 2-(aminomethyl)benzimidazole. In summary, this is the first study to describe an efficient method of obtaining a series of novel tetradentate semicarbazone ligands and nickel complexes with benzimidazole and acylhydrazone moieties from the synthesized precursors, which were derived from acid hydrolysis of sydnone compounds.
